# Soft 3D electromagnetic structures with rapid, complex shape morphing

**DOI:** 10.1126/sciadv.aea5264

**Published:** 2025-11-28

**Authors:** Jeonhyeong Park, Qifeng Lu, Ben Jeffery, Heling Wang, Xinchen Ni

**Affiliations:** ^1^ Department of Mechanical Engineering, The University of Texas at Dallas, Richardson, TX 75080, USA.; ^2^ Department of Engineering Mechanics, Zhejiang University, Hangzhou 310027, China.; ^3^ Department of Materials Science and Engineering, Northwestern University, Evanston, IL 60208, USA.; ^4^ Department of Mechanical Engineering, Northwestern University, Evanston, IL 60208, USA.; ^5^ Department of Civil and Environmental Engineering, Northwestern University, Evanston, IL 60208, USA.

## Abstract

Soft 3D systems capable of dynamic, real-time shape morphing have broad applications in flexible electronics, biomedical devices, and soft robotics. Existing methods typically rely on stress relaxation in prestretched elastomeric substrates to transform 2D precursors into 3D structures. However, these structures lack the ability for further localized programmability after transformation. This work introduces a class of soft, 3D morphable electromagnetic structures that enable fast, reversible shape transformations, with precise local programmability even after the initial 3D transformation. These systems provide access to sophisticated geometries and motions that were previously unattainable. This approach combines controlled compressive buckling of liquid metal microfluidics and Lorentz force actuation to drive the transformation, guided by multiphysics computational modeling. A 4D electronic system serves as an application example, demonstrating the potential of these spatially and temporally programmable soft systems.

## Teaser

A class of soft systems morphs into complex 3D shapes on demand, enabling reconfigurable devices and robotics.

## INTRODUCTION

Soft shape-morphing structures that can dynamically change their shapes are ubiquitous in biology, where they perform critical functions in living organisms. These systems inspire the development of analogous man-made technologies for emerging applications in flexible electronics (*1*–*3*), soft robotics (*4*–*6*), and smart medicines (*7*–*9*). Active materials, such as liquid crystal elastomers (*10*–*15*), dielectric elastomers (*16*–*19*), hydrogels (*20*–*25*), conductive polymers (*26*, *27*), and shape-memory polymers (*28*, *29*), are often combined with engineering strategies like origami (*30*–*35*) and kirigami (*36*–*38*) to enable soft shape-morphing systems. Despite notable progress, challenges remain in programmability, shape complexity, morphing speed, and shape fixation. Specifically, many current systems struggle with precise, localized adjustment of shapes (*39*, *40*), the inability to achieve complex 3D shapes (*41*, *42*), slow response times for dynamic applications (*43*, *44*), and the ability to maintain a deformed shape without persistent external stimuli (*45*–*47*).

Here, we introduce a soft 3D electromagnetic system capable of fast, reversible shape morphing into complex configurations with programmable localized control. The system exploits the unique characteristics of liquid metal, which has predominantly been used as a passively deformable conductor (*48*–*50*), combined with mechanically guided assembly (*51*–*55*). The liquid metal is first patterned and sealed in silicone-based microfluidic channels using 2D soft lithography, followed by transformation into first-order 3D shapes using controlled compressive buckling. Applying currents through the liquid metal microchannels in the presence of a magnetic field further morphs these first-order shapes into higher-order configurations with large degrees of local deformation. Examples of nearly one dozen structures and the formation of over 50 distinct higher-order shapes, guided by finite element analysis (FEA), demonstrate this scheme’s ability to create previously unattainable sophisticated geometries. In addition, the phase transition of liquid metal enables shape fixation, resulting in freestanding 3D structures. As an application example, a 4D electronic system in the form of a 3D reconfigurable light-emitting device, whose brightness is tunable by its shape morphing, is demonstrated. The concepts introduced here offer promising applications in biomedical devices, wearable technologies, and beyond.

## RESULTS

### Concepts and morphable 3D electromagnetic structures

**Fig. 1.**
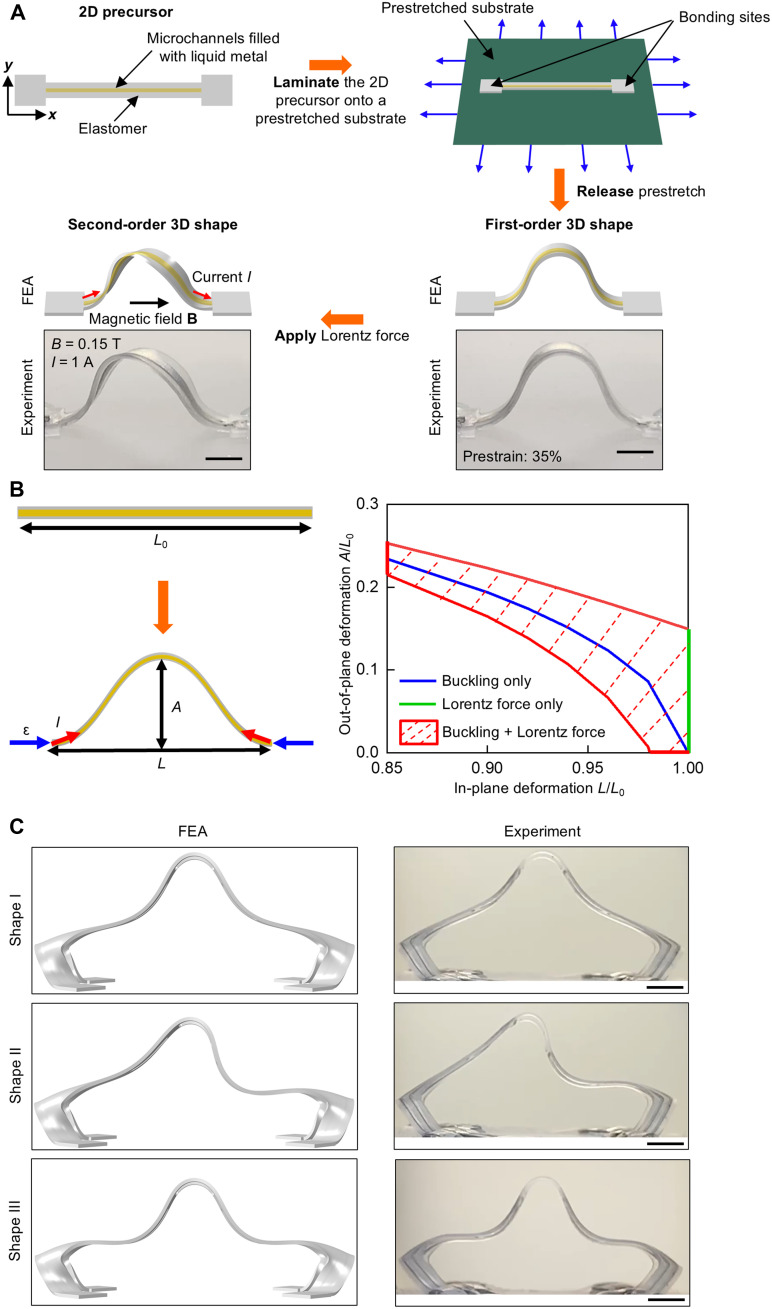
3D morphable electromagnetic structures enabled by mechanically guided assembly of liquid metal microfluidics and Lorentz force actuation. (**A**) Schematic illustration of the fabrication process and morphing mechanisms. Laminating a soft ribbon-type 2D microfluidic precursor filled with liquid metal onto a prestretched substrate at selected bonding sites and releasing the prestretch causes the 2D precursor to buckle into a first-order 3D shape. Applying a programmable Lorentz force transforms the structure into a second-order 3D shape. (**B**) Accessible deformation space of a representative ribbon structure for out-of-plane and in-plane deformations achieved through buckling, Lorentz force, and their combination. (**C**) FEA and experimental results (optical images) of three second-order shapes of a multiribbon structure that resemble the first three modes of an Euler-Bernoulli beam. Scale bars, 5 mm.

Figure 1A presents the schematic illustration of the concept and steps for forming rapidly morphable electromagnetic 3D structures, demonstrated here using a basic ribbon design. The process begins with fabricating 2D microfluidic precursors through standard soft lithography techniques. The ribbon structure incorporates microchannels (width: 300 μm and height: 200 μm) filled with eutectic gallium-indium (EGaIn; 75 wt % gallium and 25 wt % indium), enclosed within thin (~100 μm) layers of a low-modulus silicone elastomer (Dragon Skin 10, Smooth-On). Detailed fabrication procedures for these 2D microfluidic precursors are outlined in Methods. The next step involves laminating the 2D precursor onto a prestretched elastomeric substrate at selected bonding sites. Releasing the prestretched substrate transforms the 2D precursor into a first-order 3D shape. Applying an electric current (*I* = ~0.9 A) through the liquid metal microchannels in the presence of a uniform magnetic field parallel to the substrate plane (*B* = ~0.15 T) induces Lorentz forces. These forces further transform the first-order 3D shape into a complex, helically twisted ribbon configuration. Two permanent disk magnets (diameter: 76.2 mm and thickness: 12.7 mm) are positioned parallel to each other, separated by a distance of 45 mm, to generate the magnetic field used in this study (variation <20 mT within a ~20 by 20 by 20 mm^3^ central volume). Detailed experimental characterization of the field appears in fig. S1. This strategy extends to structural arrays, where the predictive nature of the FEA modeling ensures deformation uniformity across elements (fig. S2).

This approach integrates compressive buckling with programmable Lorentz forces, expanding access to previously unattainable deformation regimes. Figure 1B illustrates this concept through FEA modeling of a representative ribbon structure. The deformation space is characterized by plotting the out-of-plane deformation (the dimensionless buckled height, *A*/*L*_0_) against the in-plane deformation (the dimensionless length, *L*/*L*_0_), where *L*_0_ is the initial ribbon length, *L* is the postbuckling distance between the ribbon ends, and *A* is the buckled ribbon height. The in-plane deformation (*L*/*L*_0_) depends on the applied prestrain (ε_pre_) through the relation *L*/*L*_0_ = 1 − ε_pre_/(1 + ε_pre_), where ε_pre_/(1 + ε_pre_) is the applied compressive strain (ε_compr_) (*51*). When applying only the Lorentz force (ε_compr_ = 0, *L*/*L*_0_ = 1), the deformation space remains confined to a vertical line, represented by the green line. Similarly, compressive buckling alone produces a 1D deformation space, as shown by the blue line. Combining both methods, however, transforms the deformation space into a 2D region, outlined by the red solid boundary and enclosed by dashed lines. For a given applied prestrain, the out-of-plane deformation is continuously tunable using electronically programmable Lorentz forces. For example, at ε_pre_ = 11.1% (*L*/*L*_0_ = 0.9), the out-of-plane deformation varies from 16.5 to 22.3%. An analytical model describes this ribbon structure (fig. S3 and Supplementary Note). Good agreement between this model and FEA results validates the analytical framework and establishes a basis for inverse design. For more complex geometries, where closed-form analytical solutions are intractable, data-driven approaches (*56*) offer a route to systematic inverse design.

The interplay between 2D precursor geometries, liquid metal microchannel dimensions, applied prestrain, and bonding site locations governs the 3D buckling behavior. Figure S1 (A to C) presents the two deformation modes of a simple ribbon structure, global buckling (fig. S1A) and local collapsing (fig. S1B), alongside the critical geometrical parameters influencing the transition between them (fig. S1C). Figure S1D plots the dimensionless buckled height along the length of the ribbon for different aspect ratios (with ε_pre_ = 50%), where *x*_0_ is the undeformed coordinate along the ribbon. At small aspect ratios (e.g., *L*_0_/*W* < 8), the weight of the liquid metal has negligible effects and global buckling dominates. As the aspect ratio exceeds 8.5, the middle section of the ribbon collapses under the effect of gravity. The prestrain also influences the buckling modes. Figure S1E shows the dimensionless buckled height along the length of the ribbon for different prestrain (with *L*_0_/*W* = 8), revealing a clear transition from local collapsing to global buckling appears when the prestrain increases beyond 40%.

**Fig. 2.**
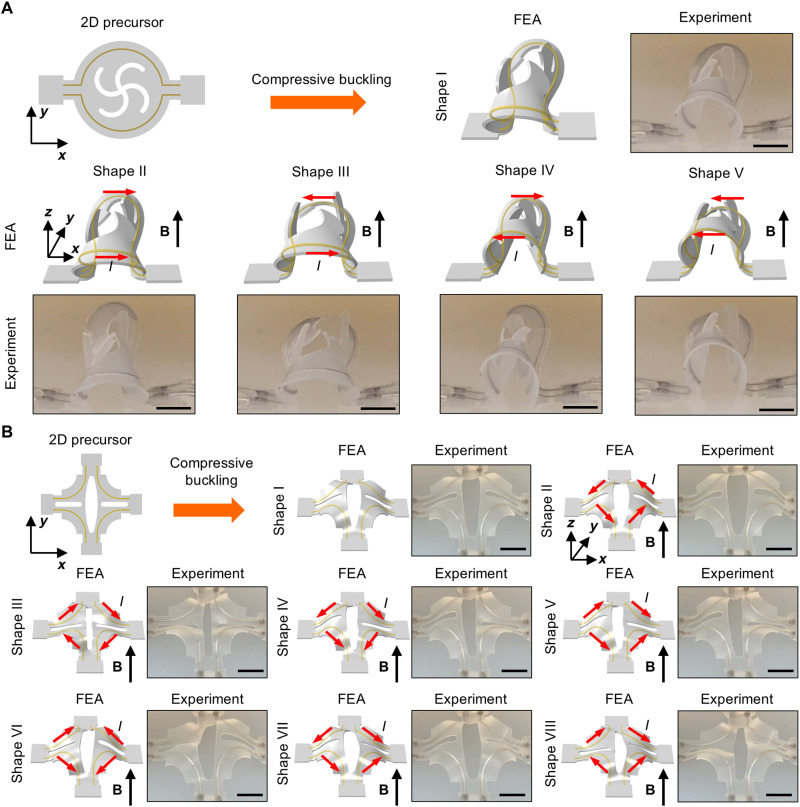
First- and second-order 3D shapes. (**A**) 2D precursors, FEA predictions, and experimental images (optical) of the first-order 3D shape and four second-order 3D shapes transformed from a circular membrane-type design with two liquid metal microchannels. Scale bars, 5 mm. (**B**) 2D precursors, FEA predictions, and experimental images (optical) of the first-order 3D shape and seven distinctive second-order 3D shapes transformed from a square membrane-type design with four liquid metal microchannels. Scale bars, 5 mm.

The design of the liquid metal microchannel pattern plays an important role in determining both the number and the complexity of accessible second-order shapes. Figure 1C presents three distinctive second-order 3D shapes of a structure composed of a central ribbon supported by a pair of side ribbons with four liquid metal microchannels, resembling the first three buckling modes of a Euler-Bernoulli beam. The 2D precursor and the corresponding applied currents appear in fig. S2. Movie S1 shows the transformation between the several second-order shapes at different currents. In contrast, a similar ribbon design with two liquid metal microchannels exhibits only the first two modes (fig. S3). Kirigami substrates (*57*) with square cut patterns (pattern dimensions appear in fig. S4, prestrain along and perpendicular to the central ribbon: 23 and 50%, respectively) are used to form the first-order 3D shapes in this example. Figure 2 presents two examples of 3D architectures obtained via compressive buckling, along with the subsequent more complex 3D shapes achieved through Lorentz force actuation, each with FEA results and corresponding optical images of experimental results. The first example (Fig. 2A) features a circular membrane with kirigami cuts in the middle and two independently addressable liquid metal microchannels. Applying currents through each of the two microchannels yields four distinctive second-order shapes, each with different levels of local buckling. The second example (Fig. 2B) features a square membrane with four concave corners and kirigami cuts at the center, incorporating four liquid metal microchannels. Symmetry considerations allow for seven distinctive second-order shapes. All 2^4^ (*16*) shapes resulting from the same current flowing in opposite directions through the four microchannels appear in fig. S5. Movies S2 and S3 show the transformations between all shapes for the two designs, including those shown here. An additional example appears in fig. S6.

**Fig. 3.**
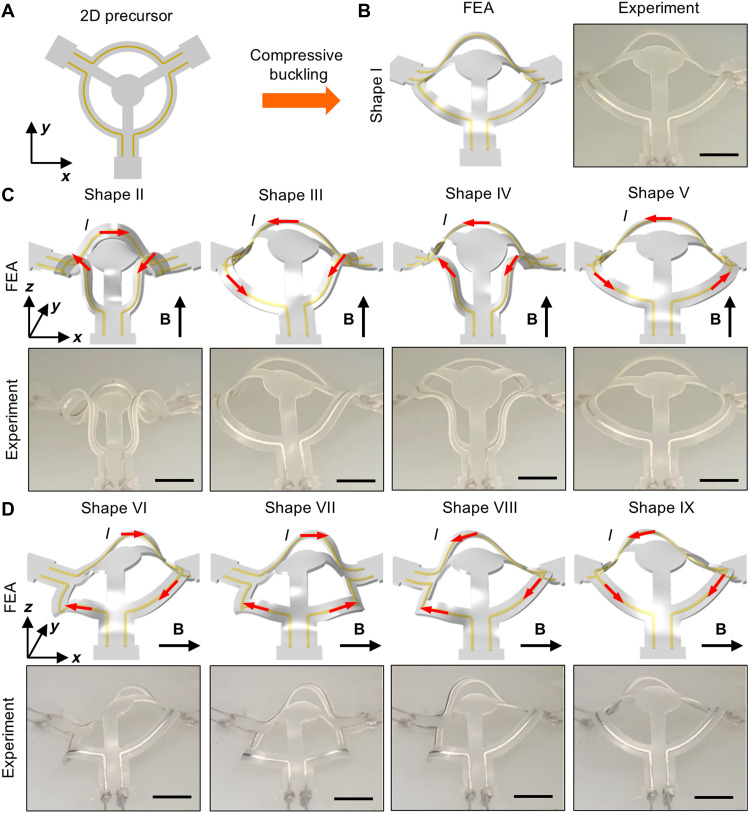
3D shapes enabled by varying external magnetic fields. (**A**) 2D precursor with a ring-type design featuring three liquid metal microchannels. (**B** to **D**) FEA predictions and experimental images (optical) of the first-order 3D shape (B) and eight second-order 3D shapes [(C) and (D)] transformed under uniform magnetic fields. Four second-order 3D shapes (C) form under a magnetic field perpendicular to the substrate plane. Four additional second-order 3D shapes (D) form under a magnetic field parallel to the substrate plane. Scale bars, 5 mm.

Varying the direction of the externally applied magnetic field enables the realization of additional shapes without altering the design. Figure 3 presents a three-channel, origami-inspired ring structure to demonstrate this approach. Compressive buckling transforms the 2D precursor into a first-order 3D shape (biaxial prestrain values are ε*_x_* = 7.5% and ε*_y_* = 10%, where the *x*-direction is left to right, the *y*-direction is bottom to top within the substrate plane, and the *z*-direction is perpendicular to it. This coordinate system is used throughout this work unless otherwise specified). When the magnetic field is aligned along the *z* axis, controlling the currents passing through the microfluidic channels results in four distinctive second-order shapes (shapes II to V), accounting for symmetrical considerations. Rotating the magnetic field by 90° to the *x* axis and applying currents in a similar manner yields four additional distinct shapes (shapes V to IX). All 16 shapes appear in fig. S7. Another example, featuring a 3D cross-shaped ribbon (ε*_x_* = 40% and ε*_y_* = 40%), appears in fig. S8. For the examples presented here, the direction of the magnetic field is manually adjusted by rotating the two permanent disk magnets. However, the strategy is compatible with electromagnetics, where the magnetic fields along all three axes can be electronically controlled with high precision, enabling further shape variations.

**Fig. 4.**
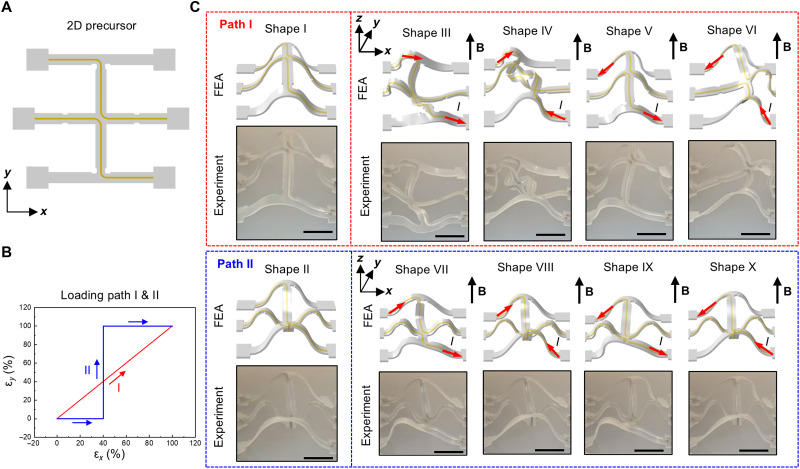
3D shapes achieved by incorporating a loading-path controlled scheme. (**A**) 2D precursor of a bi-stable multi-ribbon structure featuring two liquid metal microchannels. (**B**) Prestretch releasing sequence for two different loading paths. (**C**) FEA predictions and experimental images (optical) of the distinctive first-order 3D shape accessed through loading paths I and II, with each path leading to four second-order shapes. Scale bars, 5 mm.

This scheme is also compatible with path-controlled compressive buckling, a technique in which the order of prestrain release is controlled on a biaxially prestretched elastomer platform (*58*). This technique provides a strategy to increase the number of first-order 3D shapes derived from the same 2D precursor, thereby further expanding the accessible second-order 3D shapes. Figure 4 presents a demonstration of the integration of path-controlled compressive buckling into the current scheme, along with FEA predictions and corresponding optical images of the experimental results. The example features a multiribbon cross design with one primary ribbon oriented along the *y* axis and three supporting ribbons along the *x* axis. Guided by FEA, the substrate was prestrained by 20% in the *x* direction and 30% in the *y*-direction. Loading path I corresponds to the simultaneous release of prestrain along both the *x* and *y* axes, whereas loading path II involves a sequential process: an initial 40% release along the *x* axis, followed by a complete (100%) release along the *y* axis, and last the remaining 60% release along the *x* axis. Driven by different multistable buckling mechanisms, applying loading path I yields a first-order shape in which all four ribbon components buckle upwards (shape I). In contrast, applying loading path II results in a distinct first-order shape (shape II), where the main and middle supporting ribbons buckle downwards. Subsequent application of Lorentz forces these different first-order shapes, generates eight distinct second-order shapes: Shapes III to VI originate from shape I, while shapes VII to X arise from shape II. Despite incorporating only two liquid metal microchannels, the addition of path-controlled buckling doubles the number of achievable second-order shapes within this framework. Movie S4 shows the shape transformation between these shapes.

### State switching, shape fixation, and freestanding structures

**Fig. 5.**
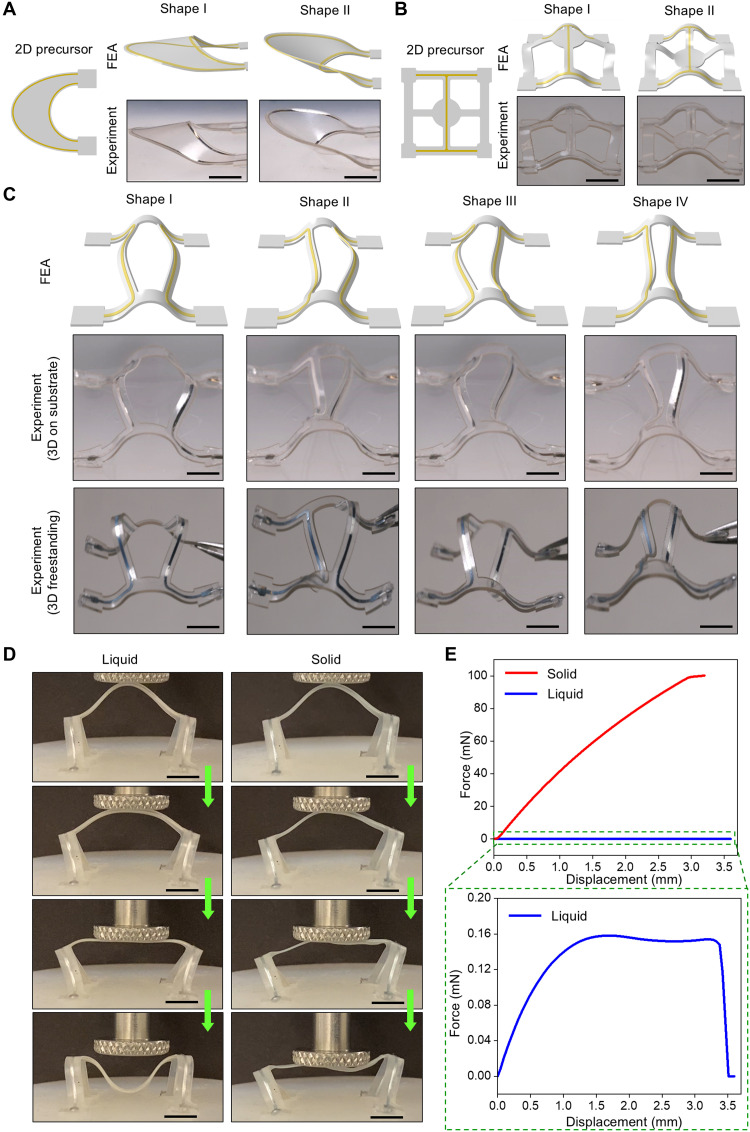
Multistable and freestanding structures. (**A**) 2D precursors, FEA results, and experimental optical images of two bistable structures with two states (shapes I and II), which can be switched reversibly by Lorentz force. (**B**) FEA results (top row) and experimental optical images of four stable configurations of a multi-ribbon structure on a substrate (middle row) and freestanding (bottom row), enabled by liquid metal phase transition. Scale bars, 3 mm. (**C**) Experimental images of the dynamic responses of a multiribbon structure under compression, with gallium in liquid and solid states. Scale bars, 5 mm. (**D**) FEA results of the force-displacement curves from compression tests. (**E**) FEA results showing the force–displacement curves from compression tests.

Previous examples require continuous application of Lorentz forces to maintain various second-order shapes. In contrast, multistable structures eliminate the need for persistent power input. In these systems, Lorentz forces only need to temporarily overcome the energy barrier separating two stable states. Figure 5 shows two representative examples of this concept: a membrane-type structure (Fig. 5A) and a hybrid membrane-ribbon structure (Fig. 5B). In both cases, shape I corresponds to the first stable state and shape II to the second. Lorentz forces act on regions where the liquid metal channels are designed on the basis of multistable buckling mechanics to enable switching between the stable states. When currents are not applied, the structure remains in its current shape due to the energy barrier between the two states. Details of the state-switching process appear in figs. S9 and S10. A more broadly applicable approach to eliminating the need for persistent external stimuli relies on using the phase-transition characteristics of liquid metal. In this demonstration, gallium (melting temperature of 29°C) is used and remains liquid at room temperature due to combined effects of Joule heating and supercooling (*59*). Figure 5C shows a double-layer, multiribbon structure, where two smaller supporting ribbons are positioned at the ends of the two main center ribbons, through which two independent liquid metal microchannels flow. This structure has four stable states. Shape I (state I) corresponds to the first-order 3D shape after compressive buckling, where both main ribbons are pushed outward. Shapes II and III correspond to configurations where one of the ribbons is pushed inward. Shape IV corresponds to the configuration where both ribbons are pushed inward. Shapes I and IV represent the open and folded states of the structure, with the overall size, measured by the distance between the midpoints of the two main ribbons, changing by approximately sixfold (from 17 to 3 mm). Lorentz forces support the transition between any of these four shapes. Applying a refrigerant (1,1,1,2-tetrafluoroethane, Thermo Fisher Scientific) to the structure while it is in one of the shapes lowers the gallium temperature to approximately −50°C through evaporative cooling, in less than 1 s. This process overcomes the supercooling effects, causing gallium to solidify and locks the structure in that particular shape. This shape fixation also facilitates the removal of the structure from the elastomer substrate, providing access to freestanding structures. The structure remains in the locked shape as long as the ambient temperature remains below 29°C. Figure 5C presents a qualitative comparison of the optical images of the 3D structures on the substrate and in their freestanding form, with no notable differences observed. The shape storage ratio (*60*), defined as 1 (representing full shape storage) minus the ratio of the change in the distance between the two main center ribbons to the original distance, is measured to be 99.8, 97.2, 96.3, and 98.1% for shapes I, II, III, and IV, respectively, confirming the robustness of the approach. Although multistable structures are used here to demonstrate the shape fixation capability, this scheme is not limited to such structures. Holding the current constant while simultaneously decreasing the temperature of the liquid metal supports the fixation of intermediate shapes. The shape fixation and resulting freestanding structures rely on the substantial stiffness change induced by the solid-liquid phase transition of liquid metal. Figure 5 (D and E) present experimental and FEA comparison of a double-layer, bistable ribbon structure under compression (compression head diameter = 10 mm) with gallium in its liquid and solid states, respectively. Movie S5 shows the different dynamic responses. In the liquid state, when the compression forces reach a critical value of ~0.16 mN, the arched-upward ribbon undergoes snap-through buckling and bend downward. In contrast, when gallium is solid, the structure loses its multistability, and the force continues to increase until the solid gallium fractures. The maximum forces in these two states differ by ~1000× (~100 mN versus ~0.16 mN).

### 4D electronics

**Fig. 6.**
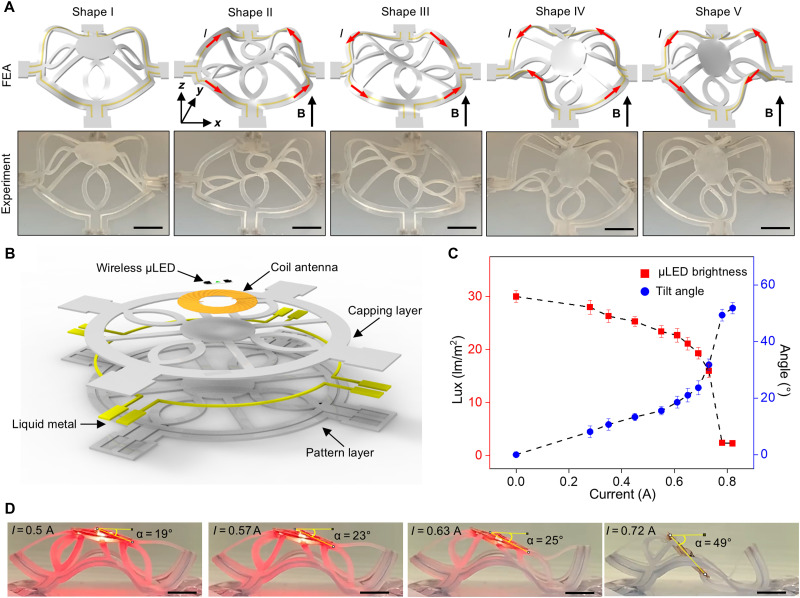
Applications of 3D morphable structures as 4D electronic systems. (**A**) FEA predictions and experimental images (optical) of the first and four representative second-order 3D shapes of a membrane/ribbon-type structure that form the basis for 4D electronics. (**B**) Exploded view illustration of the multilayer 2D precursor. (**C**) Experimental results of the tilt angle, α, of the center platform and the μLED brightness as a function of the applied current. (**D**) Experimental images (optical) showing the electronically programmable, deformation-driven tuning of the μLED brightness. Scale bars, 5 mm.

This 3D construct can also support additional functions. Figure S11 and movie S6 present an example of a 3D morphable interconnect in which the liquid metal microchannels serve as both active shape-morphing elements and functional conductors. Furthermore, this system can integrate functional components to enhance capabilities and broaden potential applications. An origami-inspired design with a circular center platform (corresponding 2D precursor shown in fig. S12) illustrates this concept. Controlled compressive buckling (ε*_x_* = ε*_y_* = 28%) lifts the center platform to a horizontal position approximately 10 mm above the substrate plane (Fig. 6A, first column). Applying maximum currents (~0.6 A) yields four second-order shapes (Fig. 6A, last four columns), tilting the center platform in any of the four directions. Movie S7 demonstrates the transformations between these second-order shapes. This shape-morphing motion has potential applications in drug delivery systems that require precise particle release. By integrating electronic components, such as wirelessly powered light-emitting systems, these 3D morphable structures transform into 4D electronic devices that dynamically change their configurations and functions. Figure 6B shows a schematic exploded view of the multilayer 2D precursor design. The design incorporates a coil antenna (8 mm in diameter) and a wireless micro–light-emitting diode (μLED) laminated onto the center platform of the liquid metal microfluidic 2D precursor. Measurements confirm the resonant frequency of the assembly to be ~13.56 MHz (fig. S13). The same compressive buckling process elevates the μLED into a horizontal orientation. Tuning the currents enables precise control of the tilt angle, α, of the platform. Activating the device involves applying radio frequency power at the coil antenna’s resonant frequency using an external loop antenna positioned ~7 mm above the structure, effectively powering the μLED. This operation relies on electromagnetic induction ( V=−dΦ/dt*),* where the induced voltage, *V*, in the receiving coil is proportional to the rate of change in magnetic flux, dΦ/dt , through the coil and, consequently, to the cosine of the tilt angle, α*.* Adjusting the currents through the liquid metal microchannels induces controlled shape morphing, altering the tilt angle and dynamically modulating μLED brightness. Figure 6C presents the experimentally measured tilt angle (right axis; blue) and μLED brightness (left axis; red) as functions of current. Incrementally increasing the current through the liquid metal channels increases the tilt angle, thereby progressively dimming the μLED. Figure 6D and movie S8 provide a visualization of this phenomenon. This electronically programmable, deformation-driven tuning mechanism is particularly relevant to applications such as light therapy, where adjustable light intensity is essential. These principles can extend to other 4D electronic devices, including advanced optoelectronic systems. Additional application examples appear in the Supplementary Materials, including a shape-reconfigurable mirror (fig. S17) and a soft gripper (fig. S18).

## DISCUSSION

In summary, this work introduces a scheme for real-time, localized shape programmability following 3D fabrication. The approach uses controlled compressive buckling to transform 2D precursors of liquid metal microfluidics into first-order 3D constructs. Lorentz force–based actuation enables independent addressing of strategically designed liquid metal microchannels embedded within the structure, allowing for rapid, reversible transformation between higher-order shapes. This system integrates advancements in materials, 3D fabrication, soft actuators, and control techniques, using rigorous multiphysics FEA modeling as a valid and effective design tool. While most demonstrations in this work are at the millimeter scale, the platform is, in principle, scalable to microscale and even nanoscale 3D liquid metal architectures. Joule heating may impose limits at very small dimensions, but these can be mitigated by design optimizations, such as serpentine geometries. The combination of magnetic field control, path-controlled buckling, and multistable structural design greatly expands the range of accessible complex 3D shapes. Shape fixation and the formation of freestanding structures are achieved through a phase transition in the liquid metal. As an application, a 4D light-emitting system with reconfigurable shape and tunable brightness, enabled by electronically programmable deformation, highlights the unique capabilities of this platform. The system’s broad compatibility with various functional materials offers diverse opportunities for 3D reconfigurable devices, soft robotics, and biomedical instruments.

## MATERIALS AND METHODS

### Fabrication of 2D liquid metal microfluidic precursors

Preparation of the 2D liquid metal microfluidic precursors began with 3D printing (printer: Form 3, Formlabs, material: Clear V4) to create a mold with patterns in the geometries of the microfluidic channels (cross-sectional width: 300 μm and height: 200 μm). Spin coating a layer of silicone elastomer (Dragon Skin 10 SLOW, Smooth-On) at 1000 rpm for 30 s onto the mold and curing at 75°C for 30 min yielded the channel layer (thickness: ~100 μm). Spin coating another layer of Dragon Skin at 3000 rpm for 30 s and curing at 75°C for 30 min formed the capping layer (thickness: ~100 μm). Both layers were treated with corona discharge for 4 min to facilitate bonding between them. Baking on a hotplate at 100°C for 40 min with some applied pressure ensured strong adhesion. A laser cutting (PLS 4.75, Universal Laser System) process defined the geometric outlines. Injecting liquid metal (EGaIn: 75 wt % gallium and 25 wt % indium) into the microfluidic channels completed the fabrication of the liquid metal 2D precursors.

### Fabrication of 3D structures

The 3D assembly process began by prestretching an elastomer substrate, prepared by spin coating and curing a silicone elastomer (Dragon Skin 10 SLOW, Smooth-On) against a flat surface. Applying a thin layer of water-soluble glue [polyvinyl alcohol (PVA) based] to the selected bonding sites and laminating the 2D precursors onto the substrate formed strong adhesion. Releasing the prestretch induced controlled buckling of the 2D precursors into 3D geometries. To create freestanding structures, immersing the fixed 3D shapes in water dissolved the PVA glue and released the structures from the substrate.

### Programmable Lorentz force actuation

A microcontroller (Arduino Mega 2560) generated control signals to adjust the current magnitude (via pulse-width modulation pins) and direction (via digital pins) through H-bridge modules powered by an external power supply (TP3005T, Tekpower). The tunable currents interacted with an external uniform magnetic field (*B* = ~0.15 T), generated by two permanent disk magnets (diameter: 76.2 mm and thickness: 12.7 mm) placed parallel to each other and separated by 45 mm, producing programmable Lorentz force actuation.

### Finite element analysis

The commercial FEA software Abaqus, combined with an in-house Python script, analyzed and predicted the nonlinear mechanical behavior of 3D structures under compressive buckling and electromagnetic actuation. Specific displacements applied to the bonding sites of the 2D precursors modeled the compressive forces induced by the prestretched elastomer substrate. A small uniform force initiated instability and was subsequently removed during the analysis. The electric module in Abaqus simulated the current density distribution, with applied voltages serving as boundary conditions. Under the specified magnetic field, a Python script calculated the Lorentz force distribution and transferred it to the Abaqus mechanics module as body forces to predict deformation. Simulations using a uniform magnetic field and those incorporating the experimentally measured nonuniform field yielded minimal differences (fig. S19). Therefore, a uniform field assumption was adopted throughout the study. The simulation proceeded in incremental steps, with approximately 10% of the total electric current added in each step. The Abaqus electric module simulated the current density distribution, with applied voltages serving as boundary conditions. A custom Python script extracted the current density and centroid coordinates for each element. On the basis of the external magnetic field, the Lorentz force distribution was calculated and transferred into the Abaqus mechanical module as body forces to predict the resulting deformation. The deformed configuration was carried forward to the next step using Abaqus restart analysis capability and used to compute the updated Lorentz force distribution. Iteration continued until the full voltage load was applied, approximating the actual physical process. Details of this simulation workflow appear in fig. S20. The walls of the microchannels were modeled by eight-node solid elements. The liquid metal was modeled as an incompressible material with negligible resistance to deformation. A refined mesh with feature sizes smaller than 1/5 of the channel wall thickness ensured accuracy. The Mooney-Rivlin hyperelastic constitutive model was adopted by all materials, with the elastic modulus (*E*) and Poisson’s ratio (*v*) being *E* = 232 kPa and ν = 0.49 for the channel walls, *E*_liquid_ = 2 kPa and ν_liquid_ = 0.49 for the liquid metal (EGaIn and gallium), and *E*_solid_ = 9.8 GPa and ν_solid_ = 0.47 for the solid gallium. The density (ρ) of materials were ρ_metal_ = 6.25 g/cm^3^ for the liquid metal and ρ = 1.07 g/cm^3^ for the channel walls, respectively.

## Supplementary Materials

### The PDF file includes:

Supplementary Note Figs. S1 to S20 Legends for movies S1 to S8
