# Supplementary material for: Soft 3D electromagnetic structures with rapid, complex shape morphing
Source: Sci Adv. 2025 Nov 28;11(48):eaea5264. doi: 10.1126/sciadv.aea5264 (PMC12662216; doi:10.1126/sciadv.aea5264)
Supplement: Supplementary file 1 — Supplementary Note Figs. S1 to S20 Legends for movies S1 to S8 [file sciadv.aea5264_sm.pdf]

Science Advances
AAAS

Supplementary Materials for

Soft 3D electromagnetic structures with rapid, complex shape morphing

Jeonhyeong Park *et al.*

Corresponding author: Xinchen Ni, xinchen.ni@utdallas.edu

Sci. Adv. **11**, eaea5264 (2025)
DOI: 10.1126/sciadv.aea5264

The PDF file includes:

Supplementary Note
Figs. S1 to S20
Legends for movies S1 to S8

Other Supplementary Material for this manuscript includes the following:

Movies S1 to S8

Supplementary Note

Analytical solutions for a single ribbon

Fig. S3A illustrates the deformation of a single ribbon subjected to combined compressive buckling and Lorentz-force actuation. When the electric current (*I*) and magnetic field (*B*) are oriented as shown, the deformed ribbon can be approximated as an arc. A simple geometric description (Fig. S3B) and a corresponding model are established to predict the ribbon deformation.

A ribbon of initial length L_0 deforms into an arc with central angle 2θ, chord length L, radius R, and arc length S:

$$L = \frac{L_0}{1 + \varepsilon_{\text{pre}}}$$

$$R = \frac{L}{2\sin\theta}$$

$$S = 2R\theta = \frac{L\theta}{\sin\theta}$$

The tensile strain and stress in the ribbon are:

$$\varepsilon = \frac{S - L_0}{L_0} = \frac{L\theta}{L_0 \sin\theta} - 1$$

$$\sigma = E\varepsilon = E(\frac{L\theta}{L_0 \sin\theta} - 1)$$

where E is the elastic modulus.

Force equilibrium in the z-direction requires:

$$2\sigma A \sin\theta = BIL$$

where A is the cross-section area of the ribbon. Combining these relationships yields:

$$\theta - \left(1 + \varepsilon_{\text{pre}}\right)\sin\theta = \frac{BIL_0}{2EA}$$

This nonlinear equation for θ can be solved numerically, after which the other deformation variables are determined. Comparison of this analytical solution and FEA results appears in Fig. S3C.

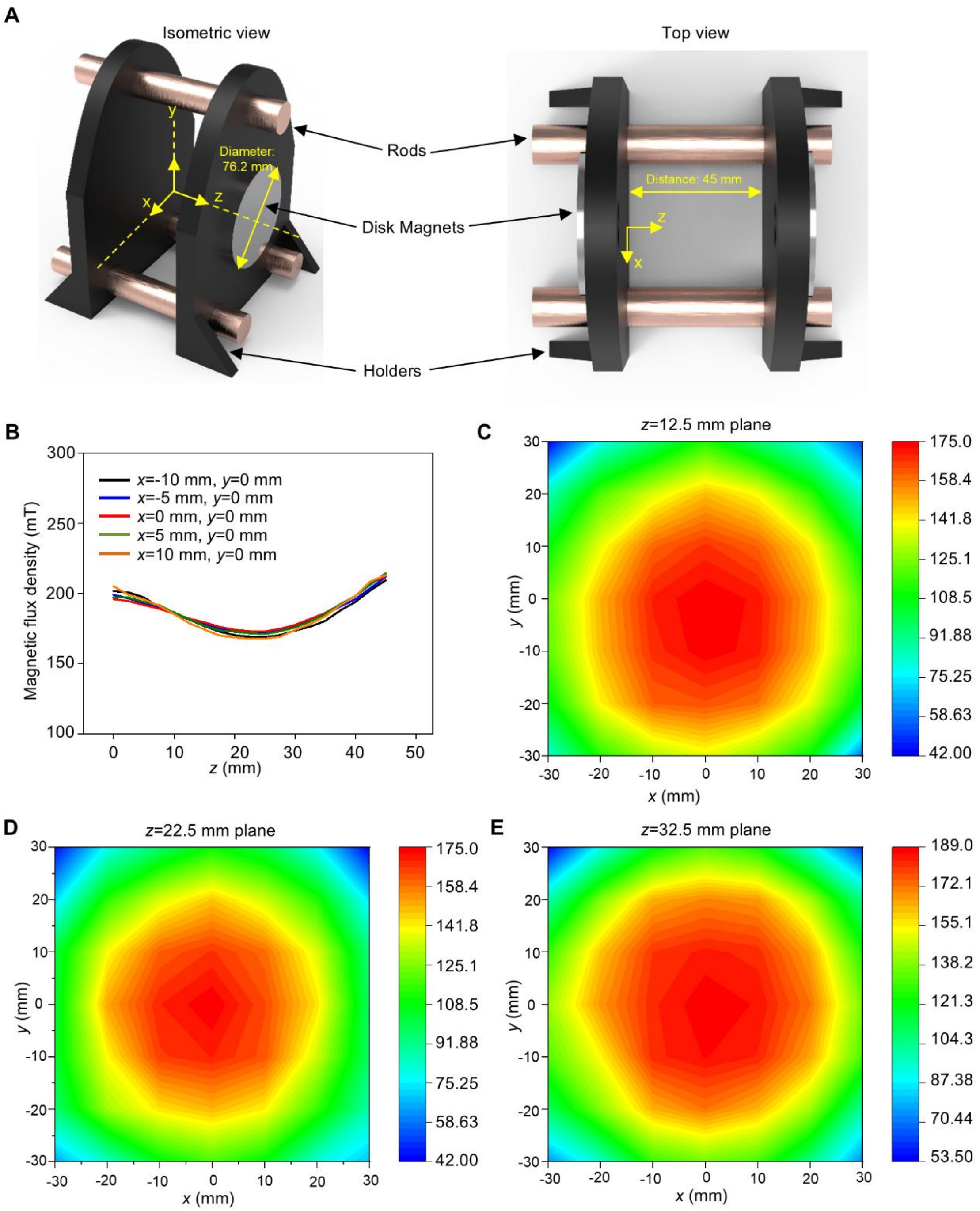

Fig. S1. Experimental characterization of the magnetic field in the parallel disk magnet setup. (**A**) Schematic illustration of the configuration with two permanent disk magnets (diameter: 76.2 mm, thickness: 12.7 mm) separated by 45 mm, with the z-axis passing through the disk centers (left disk at z = 0, right disk at z = 45 mm). (**B**) Measured magnetic flux density B_z along the z-axis at y = 0 for lateral positions x = –10 mm, –5 mm, 0 mm, 5 mm, and 10 mm. (**C–E**) Spatial maps of measured magnetic flux density B_z in x–y planes at z = 12.5 mm, 22.5 mm, and 32.5 mm.

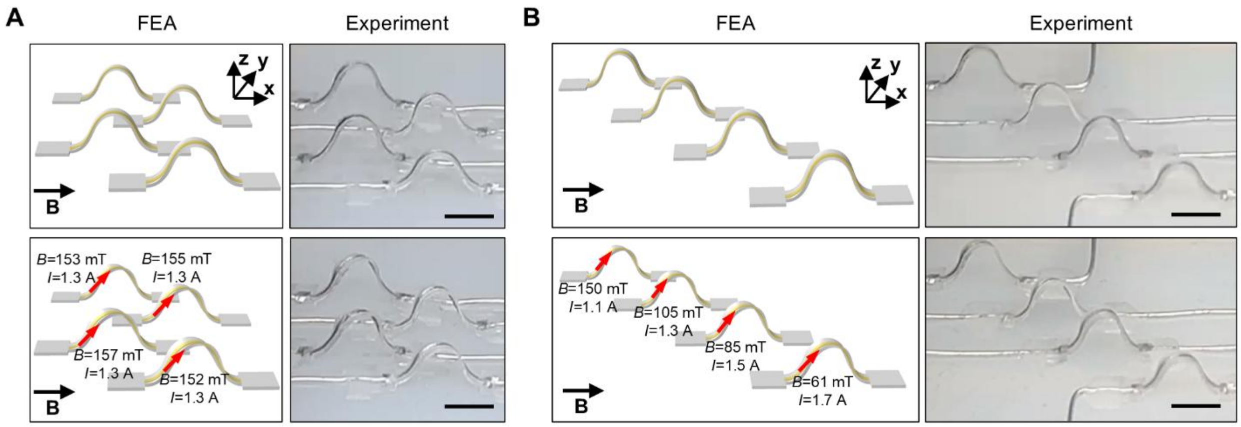

Fig. S2. Deformation uniformity in structural arrays under external magnetic fields. (**A**) FEA predictions and experimental images of a 2×2 array of ribbons within the central region ~20 × 20 × 20 mm^3) of the parallel disk magnet setup (Fig. S1), showing preserved uniformity under the same applied current despite small variations in field strength. (**B**) FEA predictions and experimental images of a 1×4 array of ribbons subjected to different local magnetic field strength, generated using a single disk magnet. Adjusted currents enable uniform deformation across all elements, demonstrating the applicability of this strategy to arrayed structures. All field strengths refer to the dominant component, B_x in this setup. Scale bars, 5 mm.

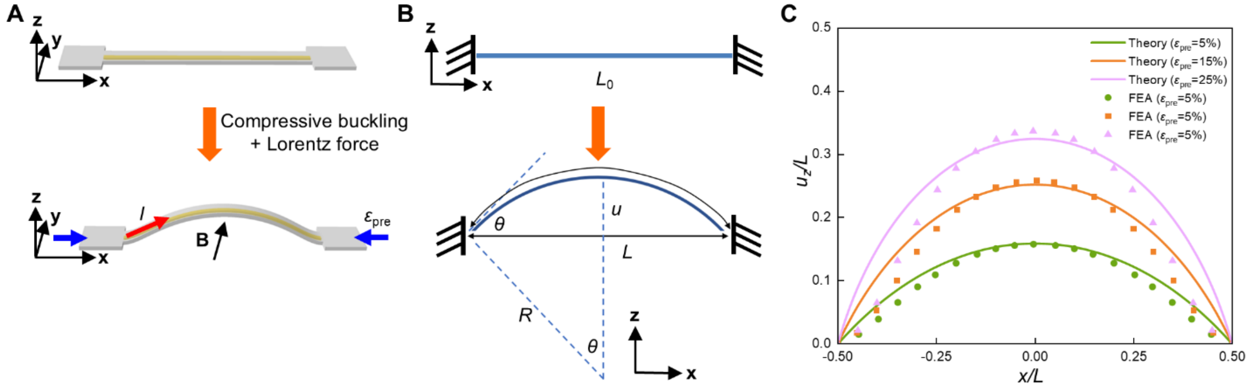

Fig. S3. Analytical model and FEA results for a single ribbon under combined compressive buckling and Lorentz-force actuation. (**A**) Schematic illustration of the ribbon structure. (**B**) Idealized model showing the geometry of the undeformed (straight) and deformed (arc) ribbon, with key parameters indicated. (**C**) Comparison of analytical solution (Supplementary Note) and FEA results under various prestrains. Agreement between theory and simulation validates the model and establishes the basis for inverse design.

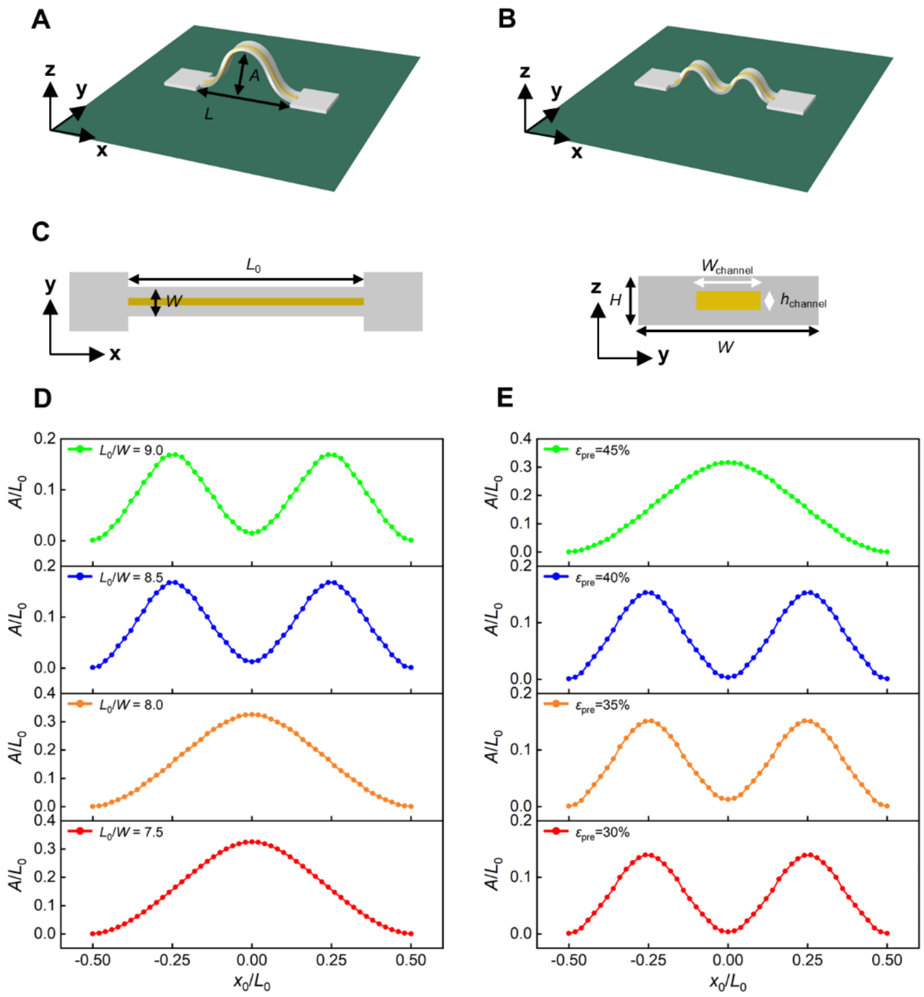

Fig. S4. Influence of geometrical parameters and substrate prestrain on the buckling mechanics. (**A–C**) Schematic illustration of two distinct buckling modes in a 3D liquid metal ribbon formed via compressive buckling—global buckling (**A**) and local collapsing (**B**)—and definition of key geometrical parameters (**C**). (**D**, **E**) FEA results showing (**D**) the effect of ribbon aspect ratio (L_0/W) and (**E**) substrate prestrain (ε_{pre}) on the dimensionless buckled height (A/L_0) along the length of the ribbon. x_0/L_0 is the normalized undeformed coordinate.

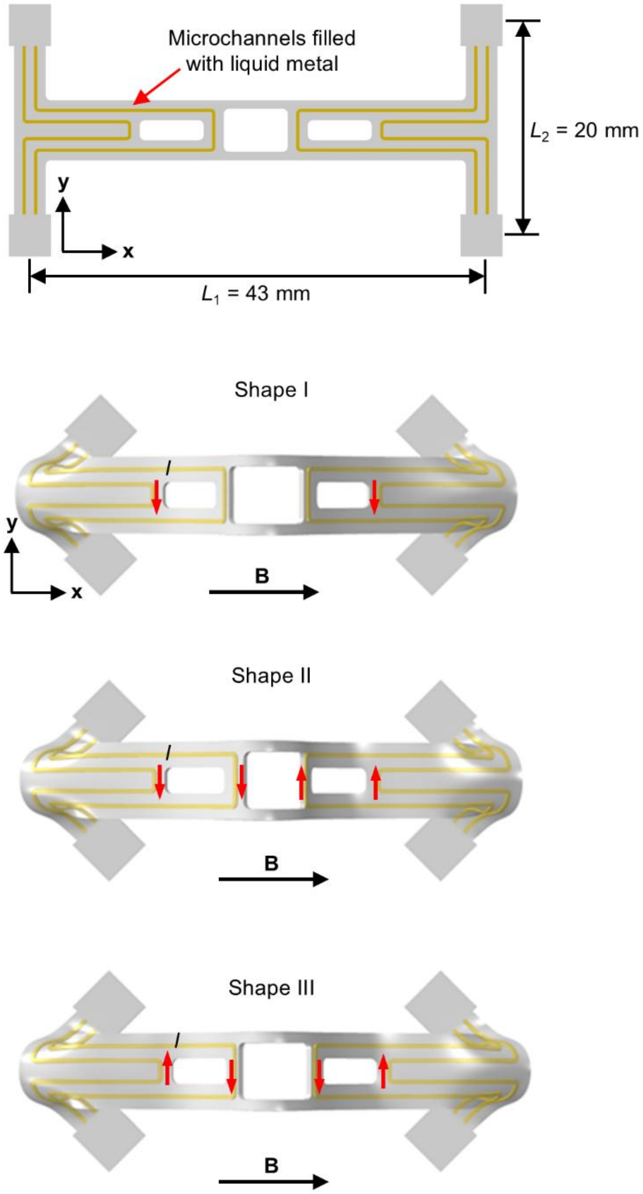

Fig. S5. 2D precursor and the applied currents of the multi-ribbon structure shown in Fig. 1C.

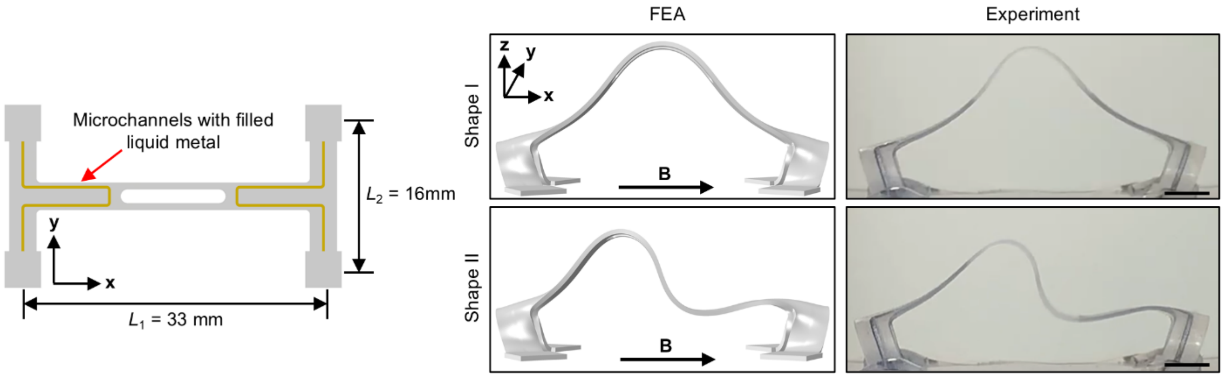

Fig. S6. 2D precursor, FEA prediction, and experimental results (optical) of the second-order shapes of a multi-ribbon structure with 2 liquid metal microchannels that resemble the first 2 modes of an Euler-Bernoulli beam. Scale bars, 5 mm.

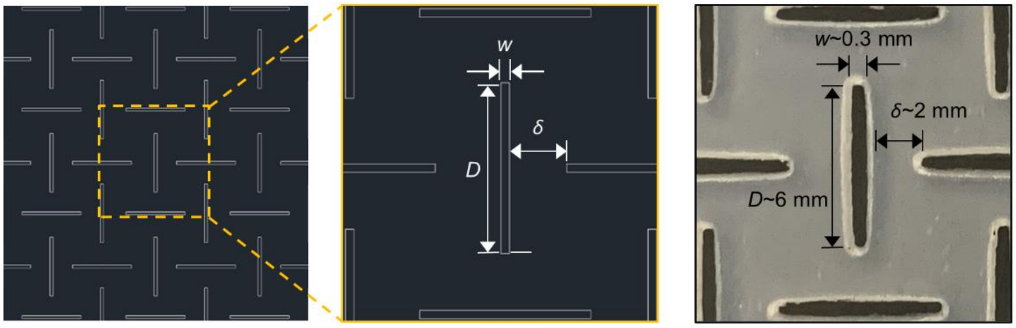

Fig. S7. Schematic diagram and experimental image (optical) illustrating the geometric parameters used in the Kirigami substrate for the multi-ribbon structure shown in Fig. 1C.

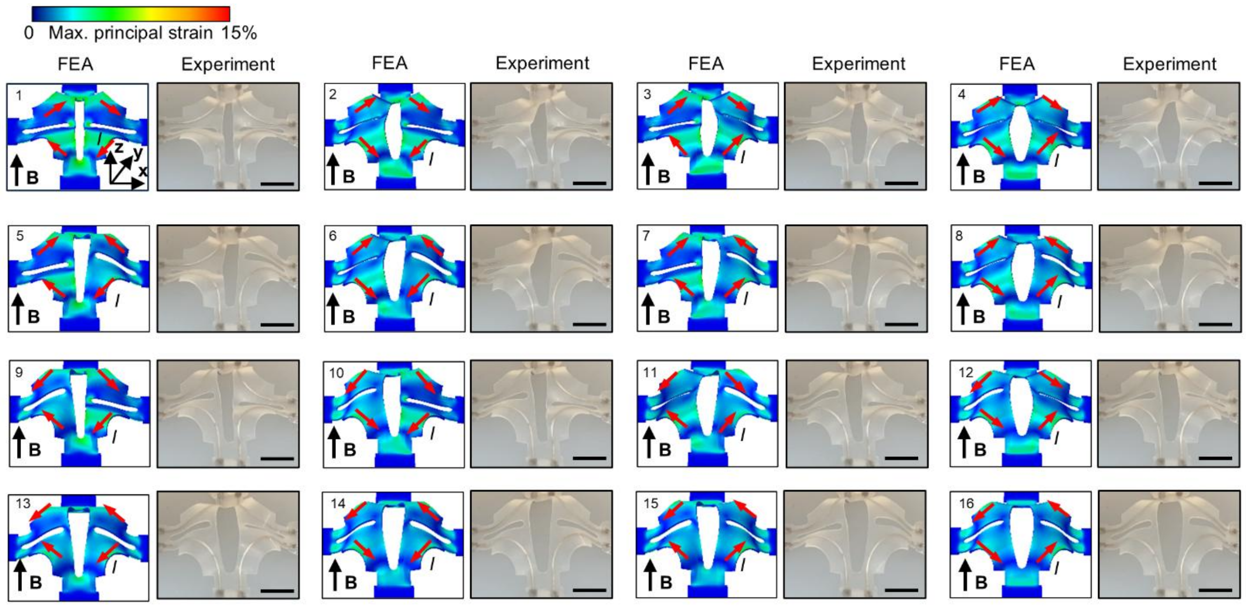

Fig. S8. FEA predictions and experimental images (optical) of the 16 second-order 3D shapes transformed from a square membrane-type design with 4 liquid metal microchannels. Color contours represent maximum principal strain. Scale bars, 5 mm.

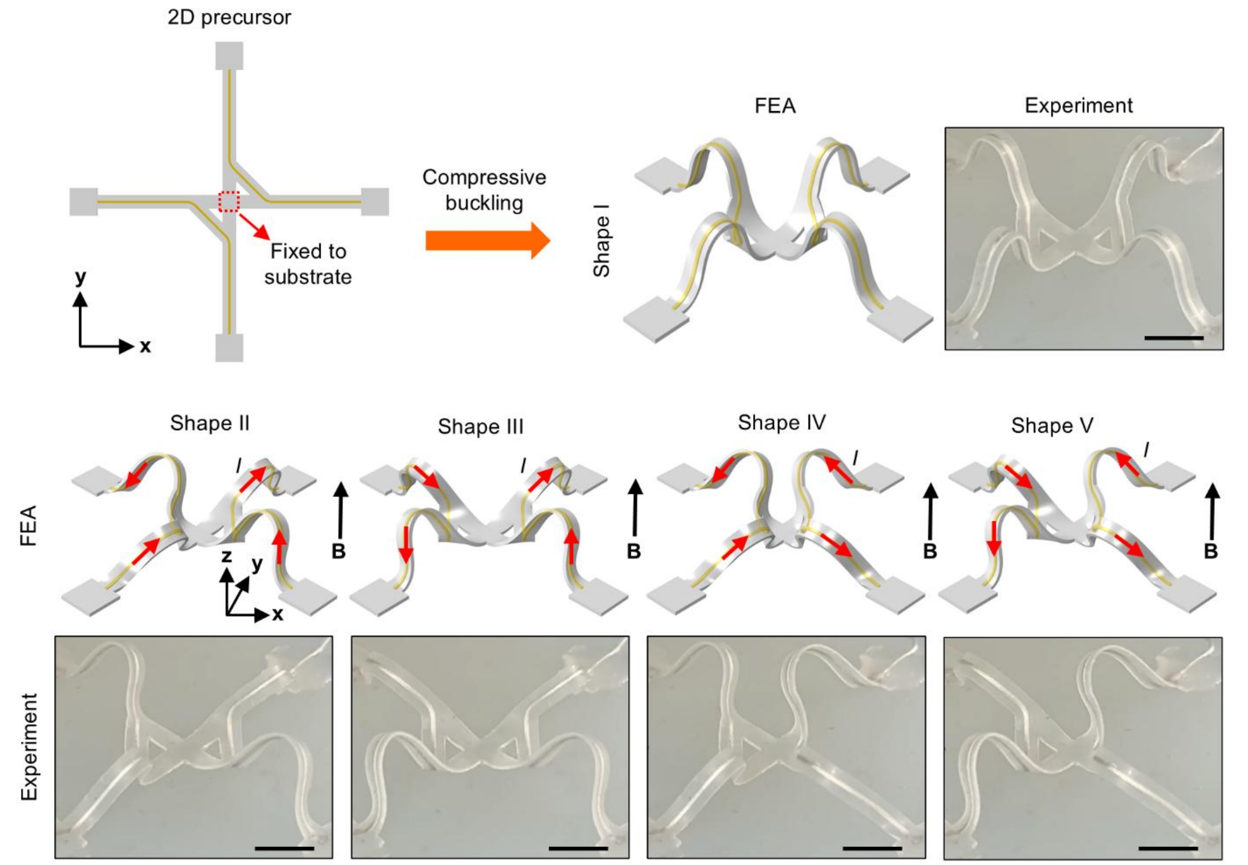

Fig. S9. 2D precursor, FEA predictions, and experimental images (optical) of the first-order 3D shape and 4 second-order 3D shapes transformed from a cross-ribbon design with 2 liquid metal microchannels. Scale bars 5 mm.

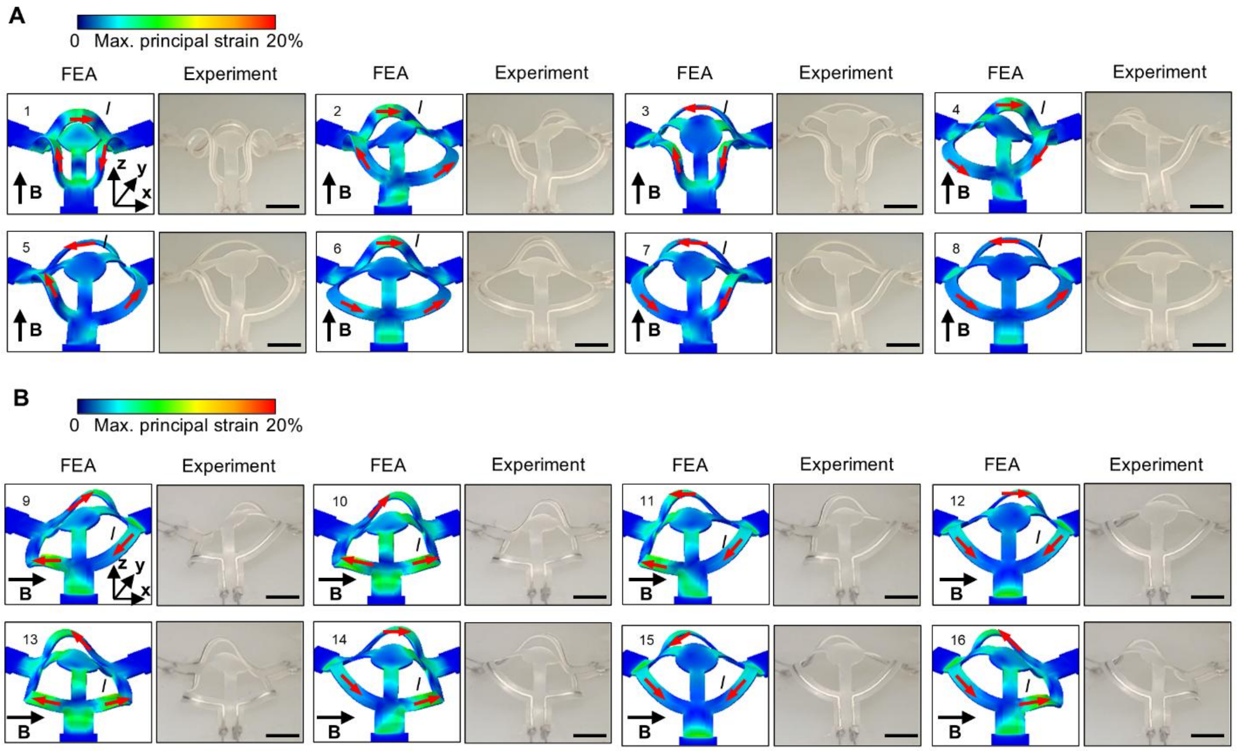

Fig. S10. 3D shapes of a square membrane-type structure enabled by varying external magnetic fields. (**A**) 8 second-order 3D shapes formed under a magnetic field perpendicular to the substrate plane (**B**) 8 second-order 3D shapes formed under a magnetic field parallel to the substrate plane. Color contours represent maximum principal strain. Scale bars, 5 mm.

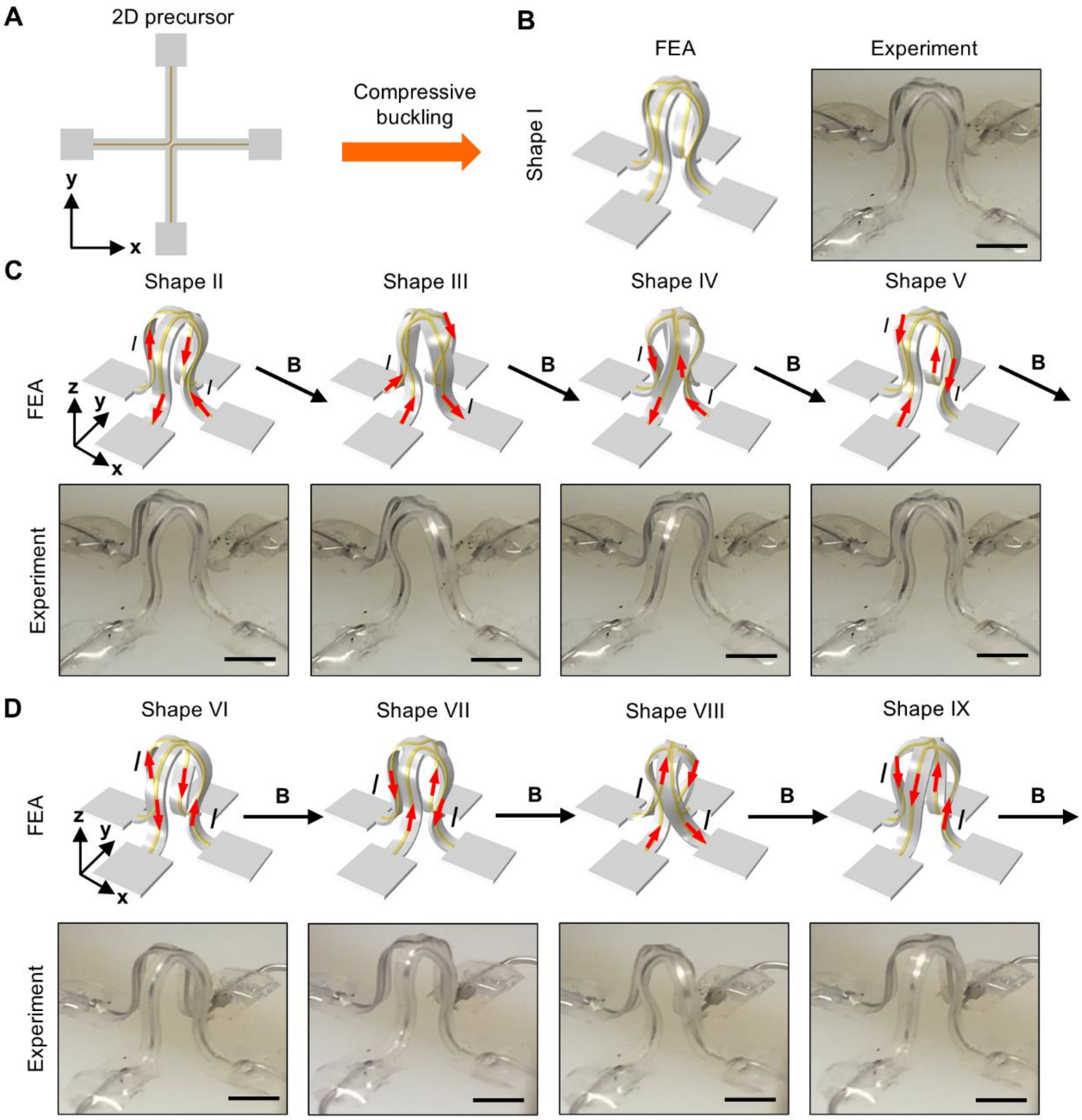

Fig. S11. 3D shapes of a cross-ribbon structure enabled by varying external magnetic fields. (**A**) 2D precursor with a cross-ribbon design featuring 2 liquid metal microchannels. (**B–D**) FEA predictions and experimental images (optical) of the first-order 3D shape (**B**) and 8 second-order 3D shapes (**C, D**) transformed under uniform magnetic fields. 4 second-order 3D shapes (**C**) form under a magnetic field parallel to the substrate plane and aligned with the axis connecting the two opposite bonding sites. 4 additional second-order 3D shapes (**D**) form under a magnetic field, also parallel to the substrate plane, but rotated 45 degrees relative to the axis connecting the opposite bonding sites. Scale bars, 5 mm.

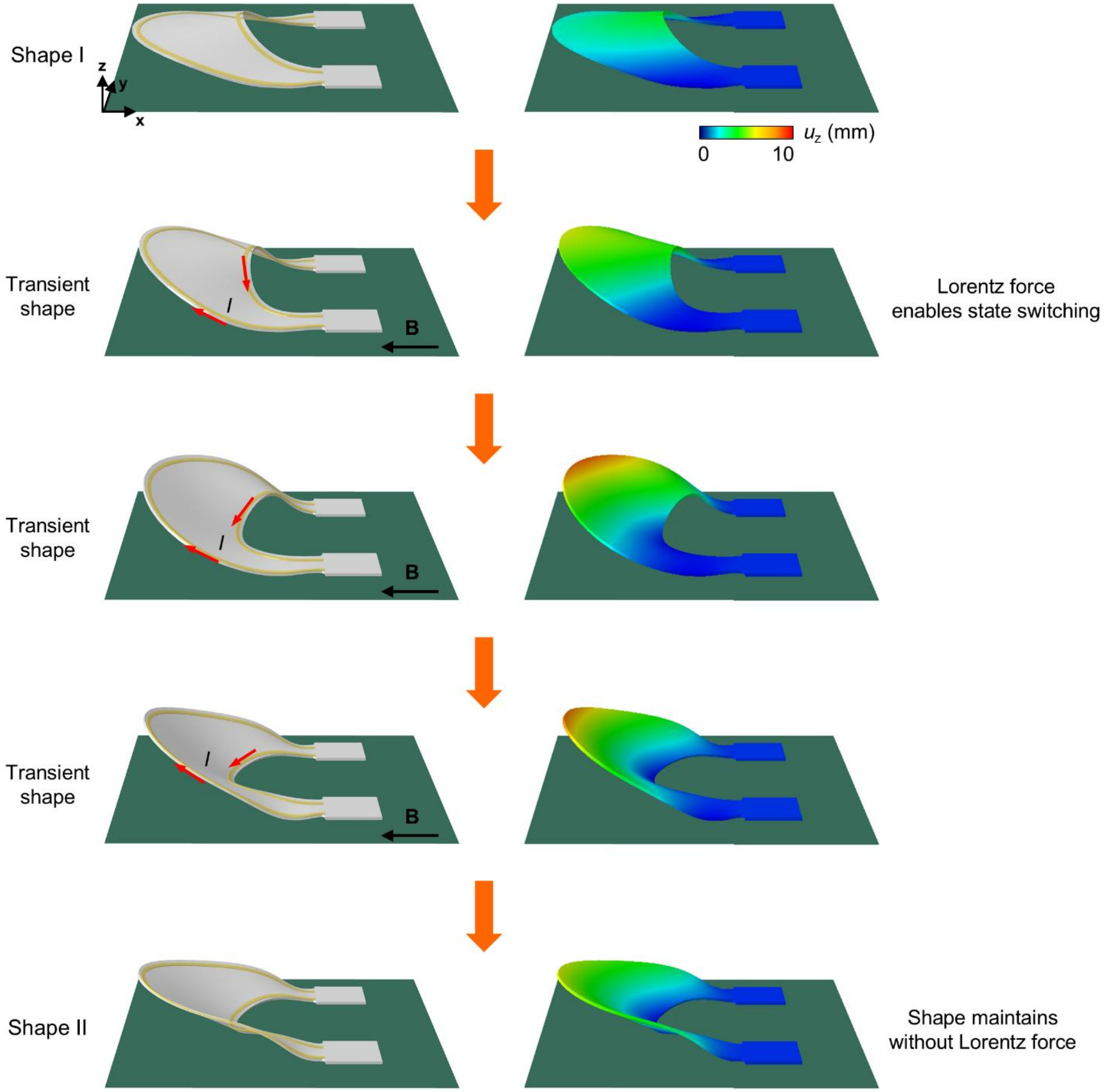

Fig. S12. Illustration of the state switching process shown in Fig. 5A via FEA results

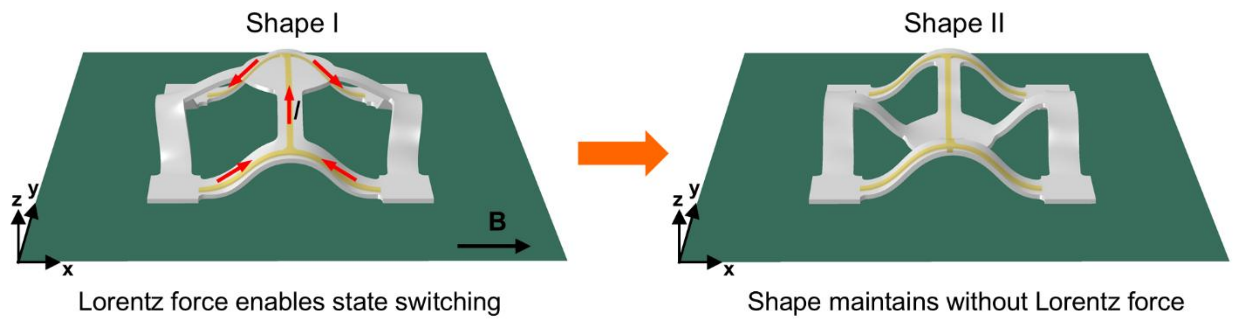

Fig. S13. Illustration of the state switching process shown in Fig. 5B via FEA results

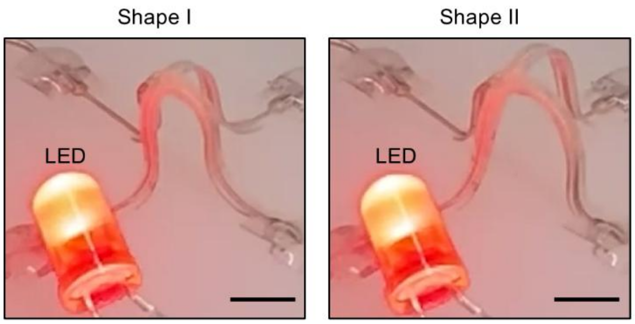

Fig. S14. A 3D morphable interconnect. The liquid metal microchannels serve as both active shape-morphing elements and functional electrical conductors for the integrated LED, enabling reconfigurable electrical and mechanical behavior. Scale bars, 5 mm.

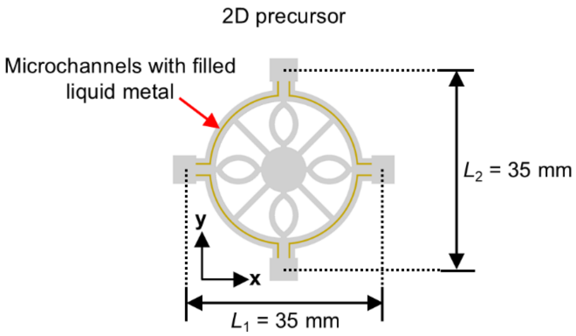

Fig. S15. 2D precursor of the membrane/ribbon-type structure shown in Fig. 6A.

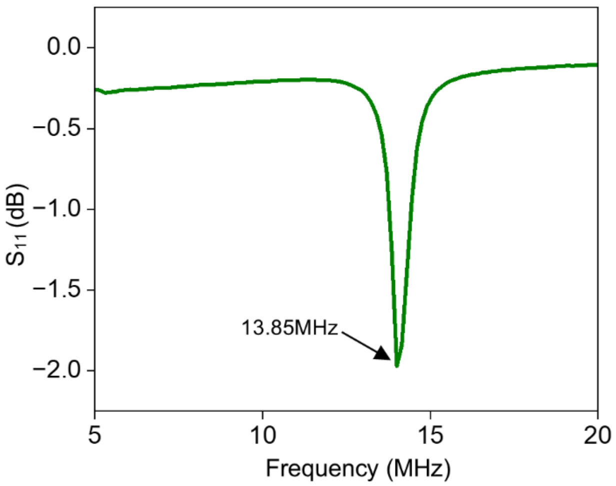

Fig. S16. Experimental measurement of the scattering parameter (S_{11}) characteristics of the μLED.

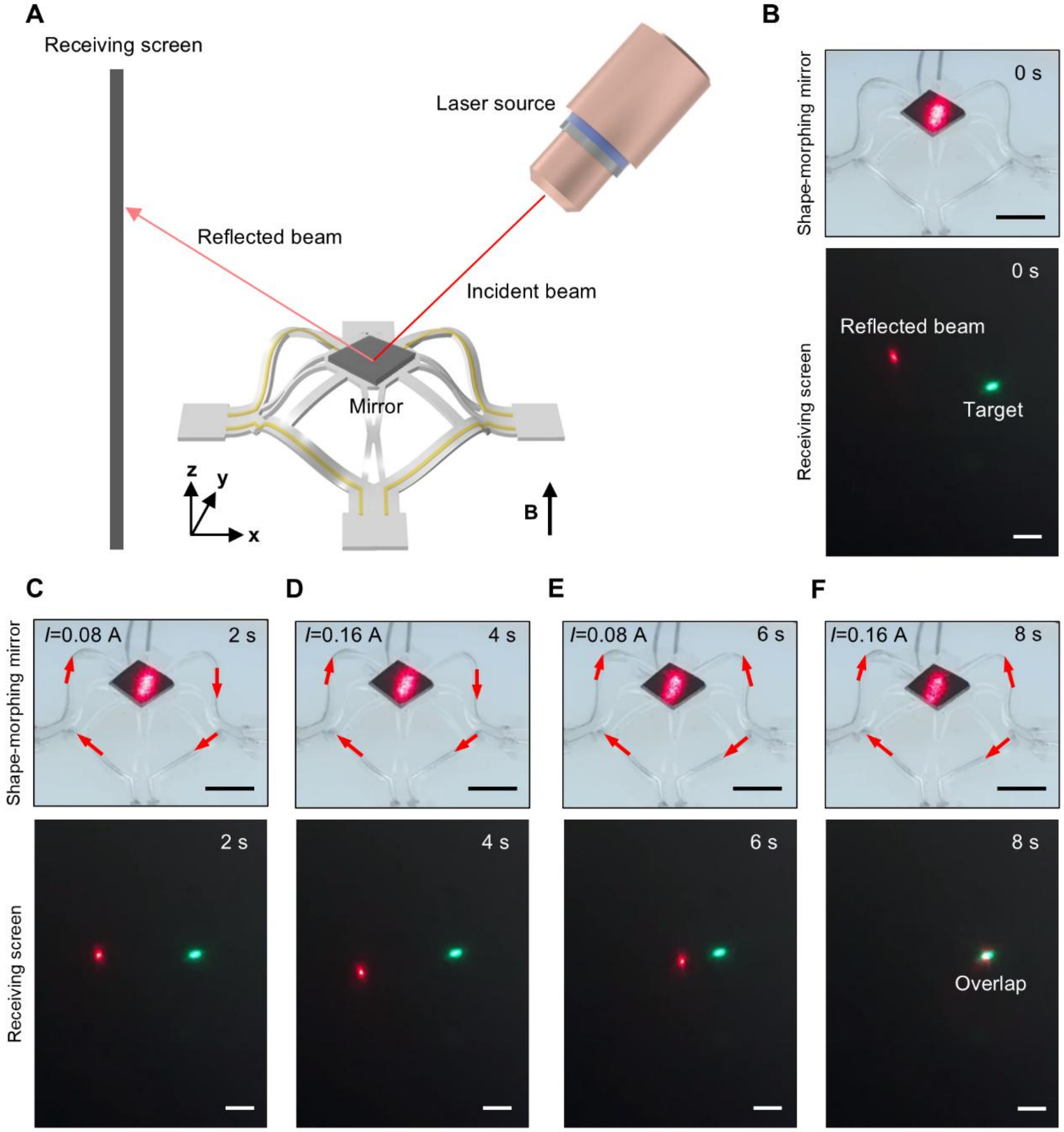

Fig. S17. Application of 3D morphable structures as a shape-reconfigurable mirror. (**A**) Schematic illustration of the mirror reflecting a red laser beam onto a screen. (**B**–**F**) Experimental images of the shape-morphing mirror and screen showing the reflected laser spot (red) steered toward the target position (green) under Lorentz-force actuation with controlled currents. Scale bars, 5 mm.

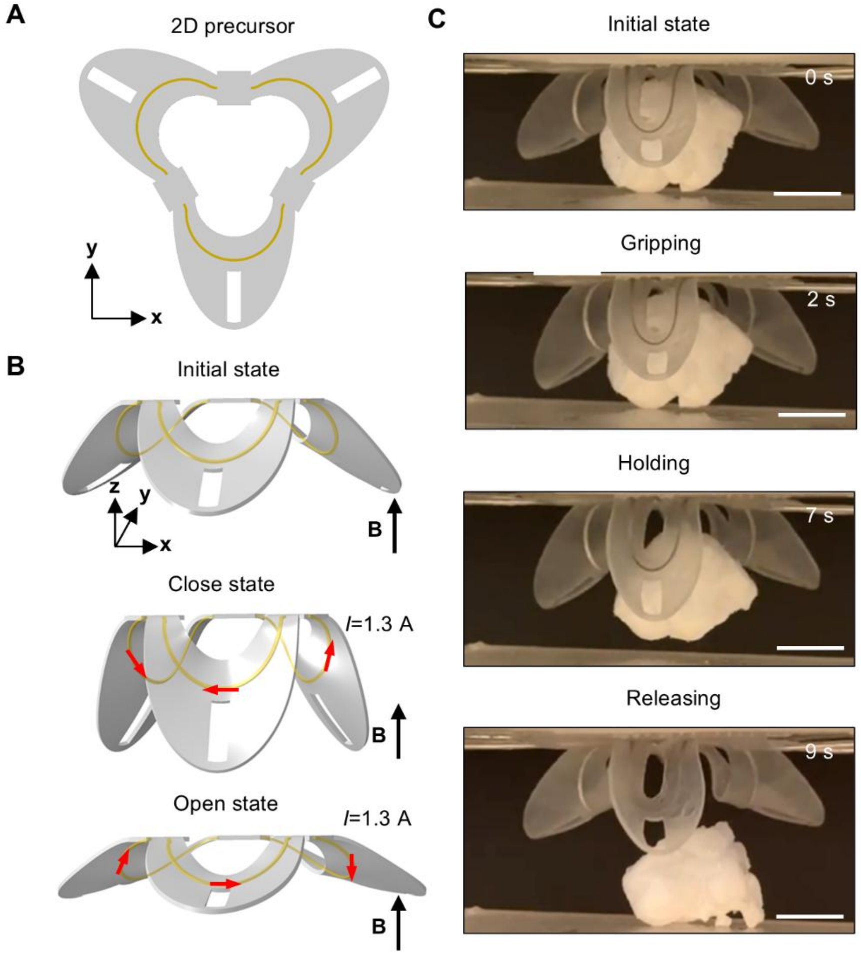

Fig. S18. Application of 3D morphable structures as a soft gripper. (**A**) 2D precursor design. (**B**) FEA results showing three functional states of the gripper achieved through first- and second-order deformations. (**C**) Experimental images showing the gripper closing to grasp and subsequently releasing an object under Lorentz-force actuation. Scale bars, 5 mm.

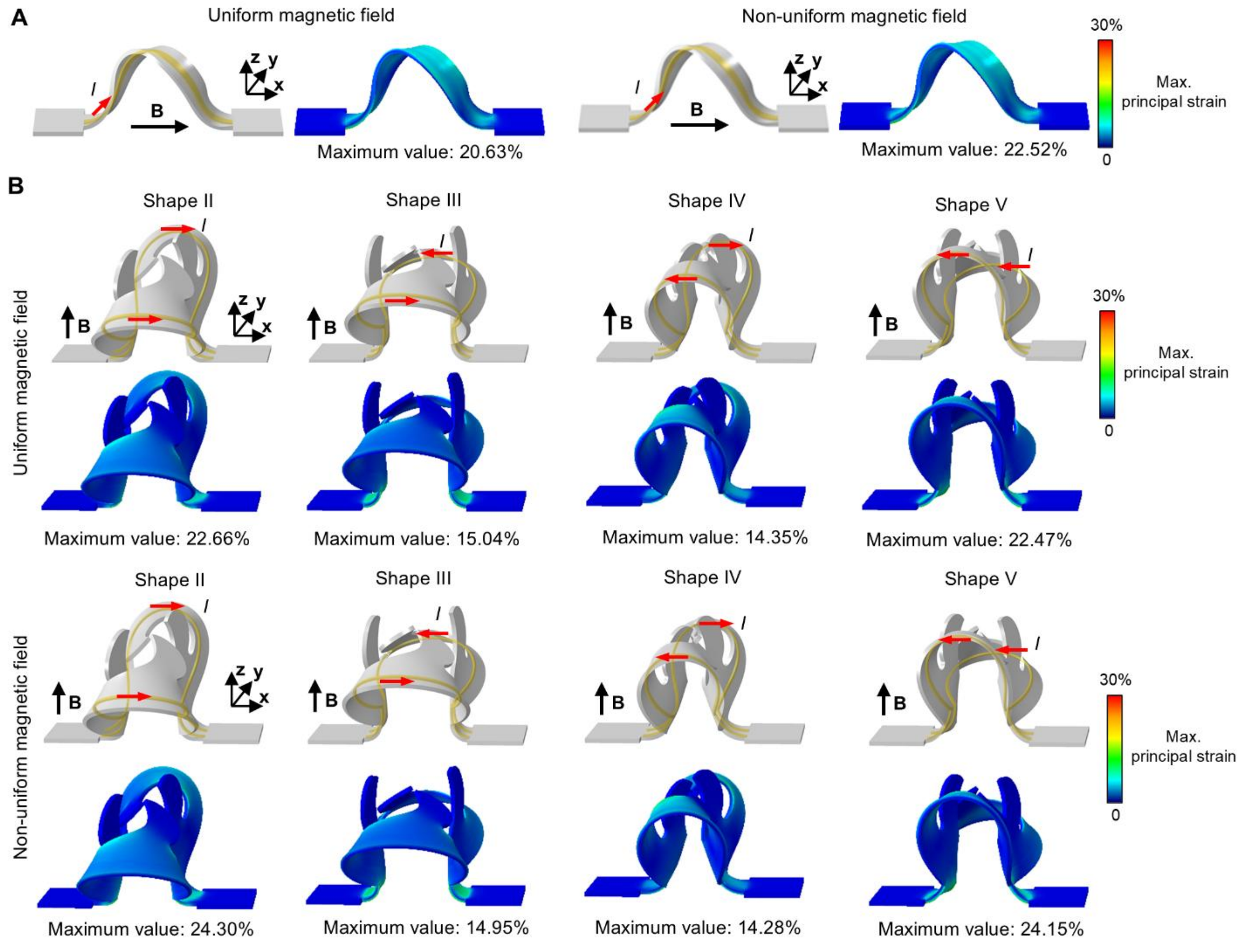

Fig. S19. FEA comparison of the influence of magnetic field non-uniformity on Lorentz-force actuation. (**A**) Single ribbon structure (as in Fig. 1A). (**B**) Circular membrane structure (as in Fig. 2A). Simulations were performed under both a uniform magnetic field (B = 0.15 T, as used in the main manuscript) and the non-uniform magnetic field distribution experimentally characterized in Fig. S1.

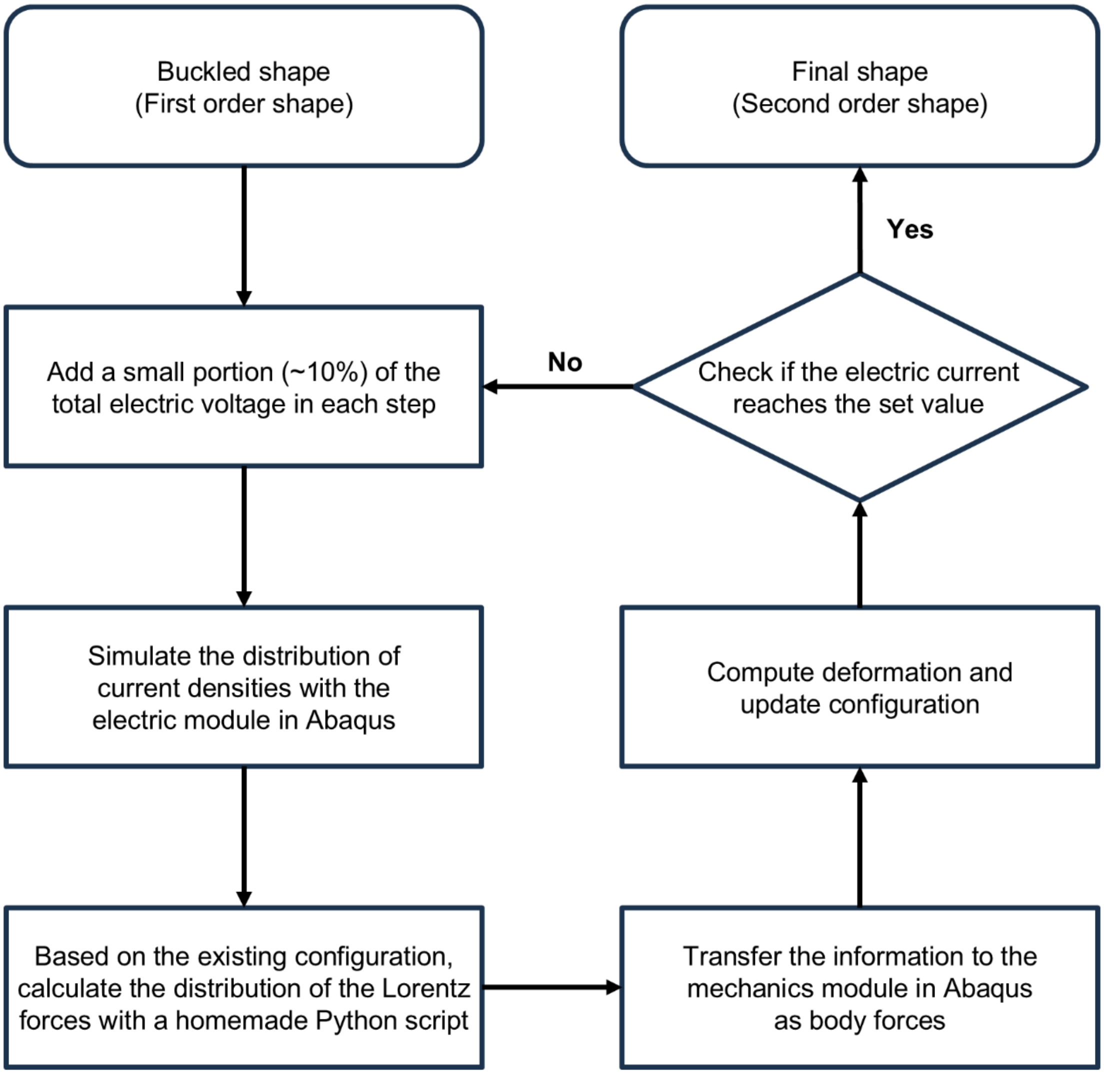

Fig. S20. Schematic workflow of the simulation process for Lorentz force–driven deformation.

Supplementary Movie Legends

Supplementary Movie 1. Shape transformations between different second-order shapes of a multi-ribbon structure with 4 liquid metal microchannels shapes including the 3 shapes as presented in Fig. 1C.

Supplementary Movie 2. Shape transformations between 4 different shapes of a circular membrane-type design with 2 liquid metal microchannels as presented in Fig. 2A.

Supplementary Movie 3. Shape transformations between 16 different shapes of a square membrane-type design with 4 liquid metal microchannels including the 7 shapes as presented in Fig. 2B.

Supplementary Movie 4. Incorporation of loading-path controlled scheme as presented in Fig. 4.

Supplementary Movie 5. The dynamic responses of a multi-ribbon structure under compression, with gallium in liquid and solid states as presented in Fig. 5C.

Supplementary Movie 6. A 3D morphable interconnect as presented in Fig. S9.

Supplementary Movie 7. Shape transformations between 16 different shapes of a membrane/ribbon-type structure with 4 liquid metal microchannels as presented in Fig. 6A.

Supplementary Movie 8. An electronically programmable, deformation driven 4D light-emitting device as presented in Fig. 6D.
